# Construction of Multiplexed Assays on Single Anisotropic Particles Using Microfluidics

**DOI:** 10.1021/acscentsci.4c02009

**Published:** 2025-01-15

**Authors:** Zengnan Wu, Yajing Zheng, Ling Lin, Gaowa Xing, Tianze Xie, Jiaxu Lin, Xiaorui Wang, Jin-Ming Lin

**Affiliations:** † Beijing Key Laboratory of Microanalytical Methods and Instrumentation, Key Laboratory of Bioorganic Phosphorus Chemistry & Chemical Biology (Ministry of Education), Department of Chemistry, Tsinghua University, Beijing 100084, China; ‡ MOE Key Laboratory of Geriatric Nutrition and Health and Department of Bioengineering, Beijing Technology and Business University, Beijing 100048, China

## Abstract

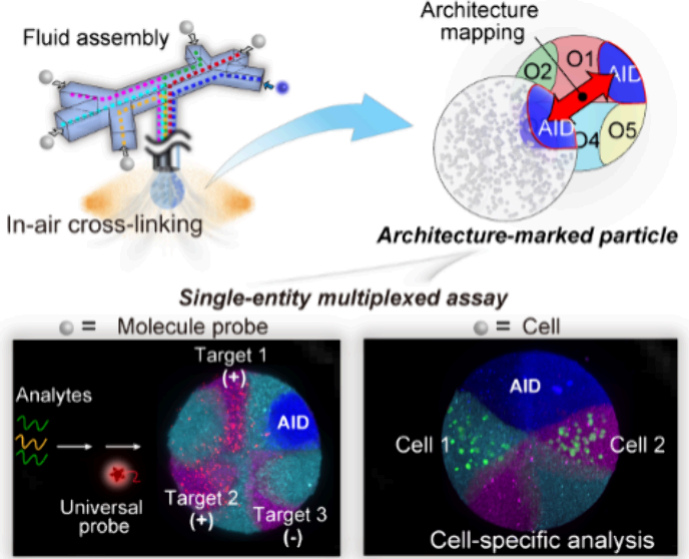

Considerable efforts have been made to develop microscale multiplexing strategies. However, challenges remain due to the difficulty in deploying functional objects and decoding high-density signals on anisotropic microcarriers. Here, we report a microfluidic method to fabricate architecture-marked anisotropic particles for performing designable multiplexed assays in a label-free manner. By controlling fluid assembly and rapid in-air cross-linking, the particles are endowed with multiple functional regions and a unique architecture identifier. The marked architecture enables an addressing mechanism that allows the profiling of embedded label-free objects by mapping a well-defined reference architecture onto the target particle. By loading analytes of interest, such as molecular probes or cells, we showed the potential of these structurally flexible particles for detecting microRNAs and studying cell interactions. The architecture-marked particles represent a new approach for single-entity assays and can be the basis for exploring more advanced microscale multiplexed applications.

## Abstract 2

A microfluidic method is reported for creating architecture-marked hydrogel particles that enable encapsulation and analysis of label-free objects, advancing single-entity multiplexed assays.

Multiplexed assays, which can simultaneously execute and monitor multiple reactions or targets in a single event, are important for a wide range of applications, including environmental monitoring,^1,2^ cell analysis,^3^ clinical diagnostics,^4,5^ and food safety.^6^ In such assays, functional experimental objects (e.g., molecular probes, cells, and enzymes) are strategically positioned on a specially designed carrier, forming a functionally integrated, analyzable system to perform specific tasks. To date, various functional carriers, such as paper,^7,8^ microfluidic chip,^9,10^ and microgel,^11−13^ have been developed. Among these, chip-based strategies (lab-on-a-chip) have been widely explored; however, their fabrication typically requires complex equipment and is costly. Paper-based methods are simple and accessible, but still face challenges in high-throughput production. Therefore, there remains a strong need for the development of designable, high-throughput, and easily accessible multiplexed platforms.

Herein, we propose the construction of multiplexed assays on a single particle-based system. Due to the advantage of their small size, high throughput, and cost-effectiveness,^14−16^ engineered hydrogel particles have propelled innovations in many fields, including tissue engineering,^17,18^ multienzyme tandem reaction,^19−21^ and multiplexed detection.^22^ However, the full potential of microparticle platforms remains unrealized primarily due to challenges in coordinating their functionalization and analyzability.

Generally, the functionalization of microparticles requires a precise arrangement of experimental objects within their architecture. For example, encapsulating magnetic materials in the core enables directional migration,^23^ while placement in a hemisphere adds rotation function.^24^ To this end, various strategies have been explored to achieve programmable spatiotemporal control of particle-based systems (lab-on-a-particle).^25−29^ For example, self-folding polyhedra enable autonomous assembly into specific configurations, but face challenges in scalability and environmental sensitivity. Microdroplet technologies offer precise chemical control within droplets but require complex and resource-intensive generation methods. Similarly, 3D printing provides customizable particle architectures but is limited by material constraints, biocompatibility issues, and process complexity. Against this backdrop, microfluidic-derived methods (e.g., microfluidics,^30−32^ flow lithography,^33^ and electrohydrodynamic^34,35^) have emerged as promising approaches, leveraging high-throughput and stable fluid manipulation to precisely arrange chemical components within diverse particle templates. However, limitations in design flexibility and biocompatibility remain challenges. More importantly, although anisotropic particle architectures hold promise for advanced applications, the consequent complex deciphering procedures impede these achievements.

For multiplexing, a prerequisite for system decoding is to differentiate signals generated by different reactions or functional objects.^36−39^ Many spatial labeling strategies require specific particle patterns, often repetitive and uniform, or rely on fluorescent tagging; however, these methods face challenges when applied to the identification of objects encapsulated within flexibly designed subregions of a single suspended particle. For instance, many efforts have been made to develop cell-laden microparticles, but the encapsulated cells are often difficult to identify; although cellular signals can be captured by microscopic imaging, attributing these signals to individual cells with similar appearances is difficult.^40^ Additionally, the random orientation of particles during imaging obscures the spatial localization of encapsulated objects.^41^ To overcome this, most works focus on directly labeling encapsulated objects with biochemical dyes for their identity tracking,^42−44^ yet these methods may have limitations in the number of simultaneous labels, potential invasive toxicity, spectral overlap, and effective time. Therefore, new microparticle platforms that allow for the flexible loading of complex experimental objects while achieving a simple signal readout are of great interest for the advancement of multiplexed strategies.

Herein, we report a biocompatible microfluidic method for generating a class of architecture-marked particles (AMPs), which can be used as versatile microcarriers for conducting multiplexed assays on a single particle; additionally, we propose an object addressing mechanism based on AMPs that enables profiling embedded label-free objects (Figure 1). The AMPs are customizable (Figure 1A): In the microfluidic assembly, each phase carrying experimental objects (functional materials or unique architecture identifier used for addressing procedures) is injected through independent channels and then merges into a single patterned flow. Subsequently, through biocompatible gas shearing and in-air cross-linking, these experimental materials are embedded within a preset particle framework. The AMPs are addressable (Figure 1B): When a specific particle is deciphering, a well-defined reference particle that mirrors its architecture is generated using an identical microfluidic procedure. By aligning them through common architecture identifiers, the known reference architecture information is mapped onto unidentified regions, enabling deterministic signal attribution of the targets. This approach resolves the identities of target objects spatially rather than relying on traditional fluorescence labels.

**Figure 1:**
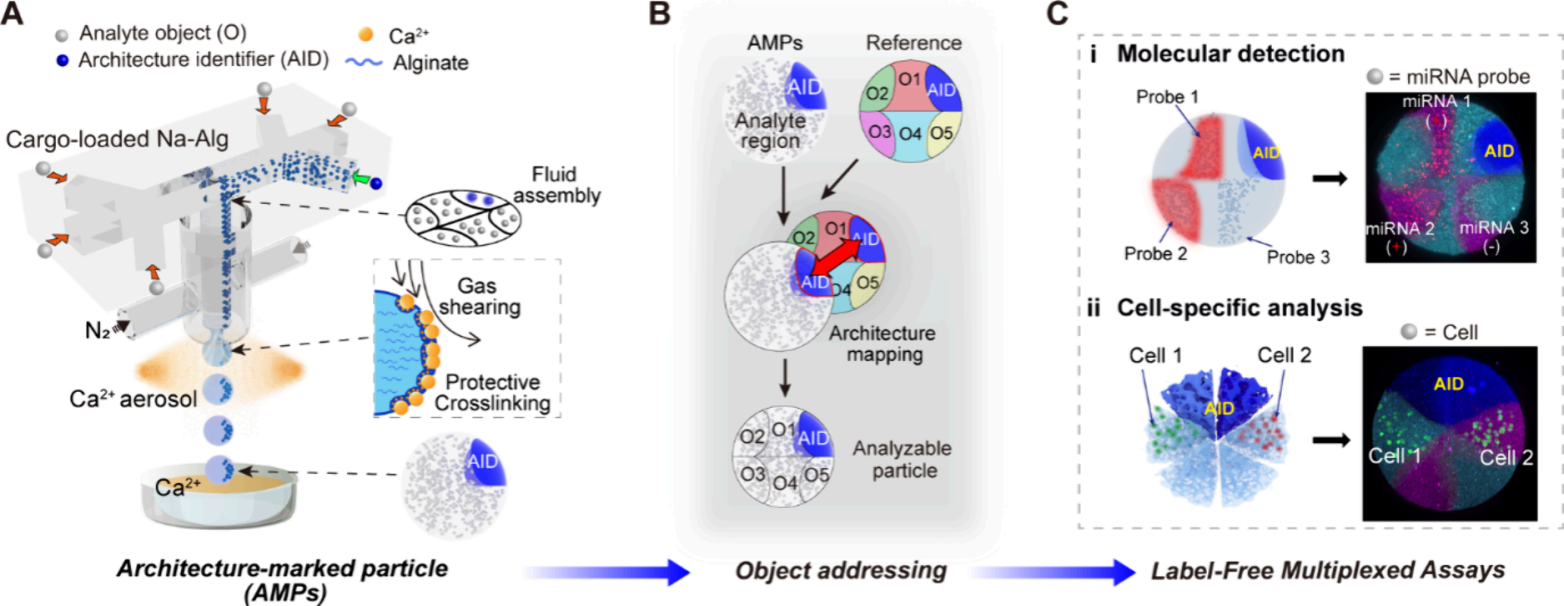
Schematic of fabrication and analysis of AMPs. (A) A microfluidic approach is used to arrange experimental objects in desired particle regions. The target particles are fabricated using recognizable architecture identifiers and various functional objects. The reference particle with an architecture identical to that of the target particle is explicitly defined. Architecture mapping enables featureless objects in the target particle to be assigned distinct identity tags. Regional abbreviations: O, object; AID, architecture identifier. (B) AMPs designed for molecular detection (i) and multicellular systems (ii) at the single-particle level.

Owing to their customizability and addressability, AMPs represent a novel approach for label-free multiplexed applications (Figure 1C). As a proof of concept, we show that analyte sensors can be integrated within user-defined particle frameworks to conduct multidetection, paving the way for microscale single-entity-based detections. Additionally, we illustrate their utility in cell culture and analysis, not only for tracking and characterizing different types of label-free cells at a population scale, but also for in situ single-cell investigations.

## Results and Discussion

### Fabrication of Particles

**Figure 2:**
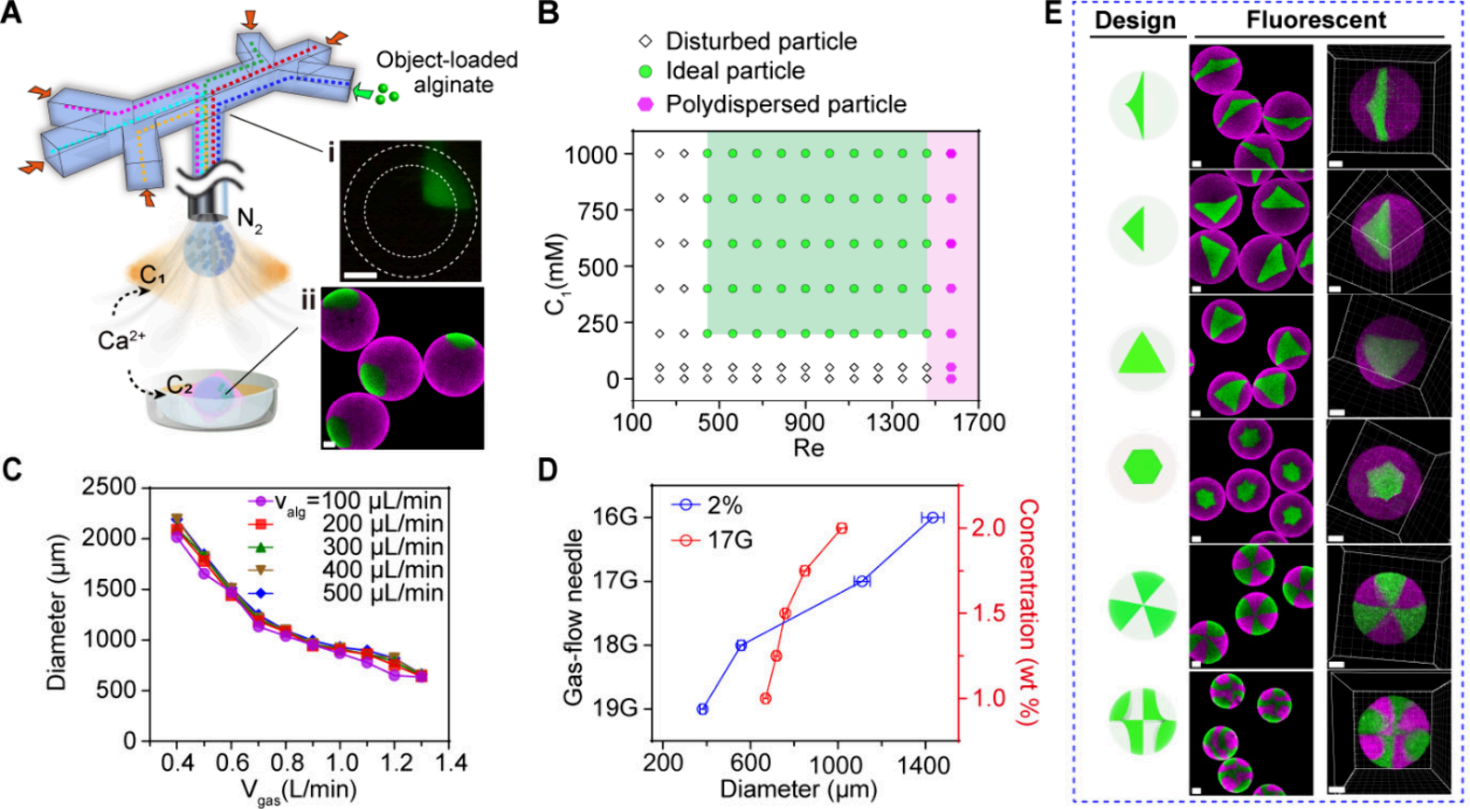
Microparticle generation. (A) Schematic of the fabrication of the particles. (i) Snapshot showing the object layout at the junction. (ii) Images of the resultant particles. Scale bar, 50 μm. (B) Optimal calcium chloride concentration for the stable formation of predesign particles. C1 is the calcium concentration of the cross-linking aerosol. Green circles represent ideal particles. White diamonds represent particles with disturbed architecture. Origin hexagon represent polydisperse particles. (C) Relationship between the particle diameter and the flow rate of alginate solution and nitrogen (*n* = 5). (D) The dependence of particle diameter on the gas-flow needle and alginate concentrations (*n* = 5). (E) Model objects are arranged in different particle architectures. Error bars represent the standard deviation. Scale bars, 100 μm.

A lab-made microfluidic device was constructed from a microfluidic channel, a droplet ejector formed by coaxial liquid–gas needles, and a solidification component containing a cross-linking solution in aerosol sprays and a receiving container (Figure 2A, Figure S1, Movie S1). The horizontal microfluidic channel was connected to the droplet ejector, forming a junction. In a typical experiment, multiple alginate fluids (a biocompatible polymer that cross-links gently in the presence of soluble calcium)^45^ carrying experimental objects were injected into the microfluidic device. Due to the low Reynolds number, these laminar flows undergo multiple rearrangements under the combined effect of channel guidance and fluid interactions to form a stable fluid layout in the microfluidic device. In this process, since the particle Reynolds number of suspended objects *R*_p_ ≪ 1 (*R*_p_ = *ρUa*(2)*/μD*_h_, where ρ is the fluid density, *U* is the fluid velocity, *a* is the object diameter, μ is the dynamic viscosity, *D*_h_ is the hydraulic diameter), their flow is dominated by viscous interactions, causing their trajectory to follow the streamlines.^46^ Therefore, the object-loaded fluid can take experimental objects to specified sites at the fluid outlet. Next, droplets can be continuously generated when the combined force of airflow shear and gravity surpasses the capillary force. Concurrently, a rapid solidification process is used to stabilize the particle layout. The abundant aerosols containing cross-linker (Ca^2+^) trigger the rapid cross-linking of the nascent droplets, instantly minimizing airflow disturbances without causing outlet blockage. The cross-linking process intensifies during droplet flight and stabilizes in the container.

To demonstrate the flexibility of assembly, model analyte objects (0.2 μm green fluorescent beads and green FBs) were planned in the edge region of the particles. Experimentally, the fluid pattern at the junction represents the particle layout (Figure 2Ai, Figure S2). Figure 2Aii shows that the resultant particles exhibit clear spatial separation without significant boundary interference, which meets the expectation. Next, we explored the optimal manufacturing parameters required to achieve the desired particle architectures. Specifically, inadequate cross-linking in the receiver solution leads to deficient mechanical properties of the hydrogel particles, inflicting structural dispersion (Figure S3). Increasing the concentration of the receiving liquid solution (*C*_2_ > 50 mM) improves the particle shape, but unexpectedly, the particle architecture remains undesired, even at an unusually high cross-linking concentration (*C*_2_ = 1M). This issue can be attributed to the inevitable internal circulation within the droplets, which disturbs the internal structure of the particle before cross-linking.^47^ To overcome this, we introduced calcium-containing aerosols at the droplet exit to initiate cross-linking as early as possible. As expected, when *C*_1_ > 200 mM, the stability of the internal structure was significantly improved (Figure S4). Additionally, airflow parameters are crucial for stabilizing the particle. Figure 2B shows that rapid in-air cross-linking (*C*_1_ > 200 mM) combined with appropriate gas parameters (Reynold number of nitrogen flow, *Re* > 445) ensures the ideal particle architecture, whereas excessive airflow (*Re* > 1461) lead to particles with uncontrollable sizes (data not shown here). Next, we focused on the adjustment of the particle size, which was found to be effectively controlled. An increase in the gas flow rate significantly reduces the particle size, while the substrate flow rate has little effect within the test range (Figure 2C). Furthermore, a positive correlation was identified between the particle size and both needle size and hydrogel concentration (Figure 2D). To optimize production efficiency, we observed that both increasing the gas flow rate and increasing the alginate flow rate significantly enhance the production frequency (Figure S5). Moreover, to explore design flexibility, the model objects were arranged into diverse particle architectures, such as triangles, hexagons, and irregularities, by adjusting the microfluidic parameters (Figure S6). In all examples, ranging from simplistic to intricate layouts, the ideal spatial-separation architectures were successfully verified by 2D and 3D imaging (Figure 2E). These results demonstrate that our microfluidic approach enables precise object arrangement in a specified particle space, facilitating subsequent functional designs.

### AMP Decoding

**Figure 3:**
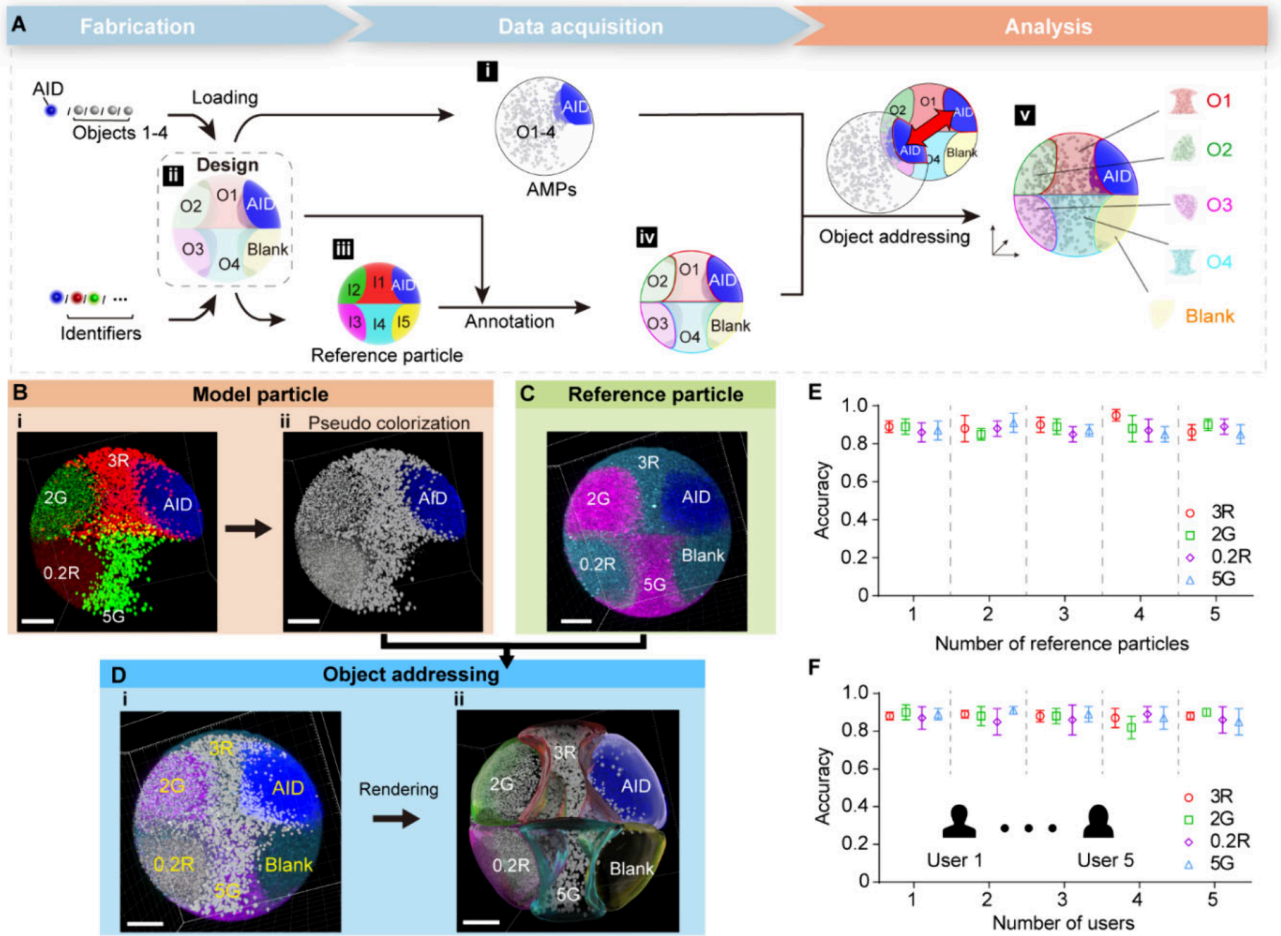
Architecture mapping for object addressing on AMPs. (A) Architecture mapping for object addressing on a particle. Conceptual description of the process of object addressing. The architecture identifier and the featureless experimental objects are arranged in the designated particle regions (i) according to the particle design (ii). The distinguishable identifiers are used to construct the reference particle (iii) according to the same design (ii). The identifiers enable visualization of the boundary of each region, and the particle design is used for the annotation of the reference particle (iv). (v) Aligning the two particles to map the spatial information on the reference particle onto the indistinguishable target particle. Regional abbreviations: O, object; I, identifier; AID, architecture identifier. (B) Fluorescence images of a model particle (i). Regional abbreviations: 2G, 2 μm green beads; 5G, 5 μm green beads; 0.2R, 0.2 μm red beads and 3R, 3 μm green beads. Pseudocolorization of the model particle (ii). (C) A fluorescence image of the reference particle. (D) Results of the particle alignment. (i) Featureless objects are assigned clear identities. (ii) 3D rendering of the fully segmented reference particle. (E) Addressing accuracy for different reference particles. The same user uses different reference particles to align 5 model particles and verify the results (*n* = 5). (F) Addressing accuracy for 5 random users (*n* = 5). Error bars represent the standard deviation. Scale bars, 100 μm.

In particle systems, nonself-identifying analyte objects are not allowed to be directly interrogated because their spatial location and regional boundaries are confusing (Figure 3Ai). However, we note that the layout of encapsulated objects in the target particle is deterministic, even though visually indistinguishable. Therefore, we assumed that by creating a reference particle to replicate the architecture and mapping its spatial information onto the target particle, it would be possible to indirectly recognize the identities of the encapsulated objects, thus facilitating downstream analysis. For this purpose, the architecture identifier (a unique visual region) is planned into the particle design (Figure 3Aii), and the identification is achieved by filling the same region of the target and reference particles with distinctive fluorescent identifiers to guide their spatial alignment. The matching reference particles are fabricated in an identical production process by replacing experimental objects with fluorescent identifiers to ensure identical architectures (Figure 3Aiii). Thus, all regions of the reference particle can be assigned identity tags according to the preset particle design (Figure 3Aiv). In analysis, the target particle was aligned to its reference, establishing a correlation between the object identities of the former and the spatial architecture information on the latter (Figure 3Av).

To demonstrate this strategy, model AMPs with an irregular layout were fabricated and subjected to identification and verification. The model AMPs were prepared using various fluorescent analyte objects (2 and 5 μm green FBs, 0.2 and 3 μm red FBs; Figure S7). The architecture identifier was designed as a region filled with 0.2 μm blue FBs. Light-sheet fluorescence microscopy was used to rapidly acquire complete particle signals (Figure 3Bi). Here, to simulate common analysis scenarios in which different target objects have similar visual appearances, all analyte objects were uniformly pseudocolorized (Figure 3Bii). This eliminates the contributions of object visual features to the identification process, rendering them indistinguishable. The reference particle was fabricated using different fluorescent identifiers (0.2 μm FBs) and was annotated according to the particle design (Figure 3C, Figure S8). Consequently, particle alignment enables the identification of ambiguous objects in the model particles (Figure 3Di). For a better illustration, the reference particle is shown in the form of 3D reconstruction, where the outlines of different colors represent the regional boundaries (Figure 3Dii; Figure S9, Movie S2).

To evaluate addressing accuracy, the original fluorescence of the analyte objects was reactivated (Figure S10 and Movie S3). The results show a good correspondence between the fluorescence signals and their given identities. Furthermore, the addressing accuracy was quantified by measuring the proportion of analysis objects that were correctly identified. Five random reference particles were tested, and the identification accuracy for all functional regions was greater than 0.85 (Figure 3E). Also, five participants were invited to evaluate this strategy, and the average addressing accuracy remained greater than 0.85 (Figure 3F). It is worth mentioning that there still exists potential for future enhancements in alignment accuracy with ongoing advancements in computer technology.

### AMPs for Multiplexed Detection

**Figure 4:**
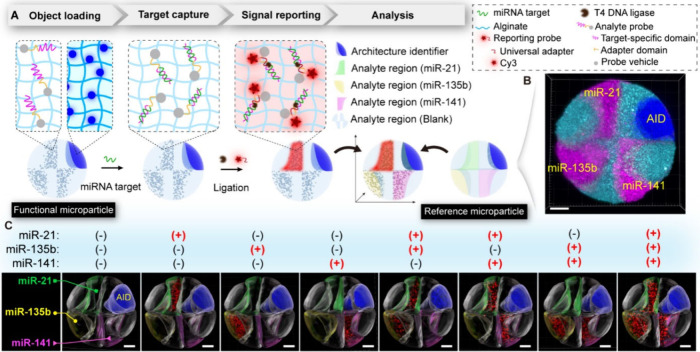
AMPs encapsulated with multiple probes as an integrated sensor: multiplexing on a particle. (A) Schematic of the multiplexed miRNA assay. Multiple miRNA targets were detected at the single particle level using specific miRNA probes and universal reporter molecules. (B) The image of the reference particle. (C) Fluorescence images of eight representative particles after different multiplexed miRNA assays. Scale bars, 100 μm.

The AMPs represent a label-free, microscale-integrated single-entity strategy that can be designed for diverse purposes. Typically, its intrinsic nature of small volume, high bandwidth, and customizability would bring about a key advancement among current multiplexed analysis methods. A microRNA (miRNA) bioassay was conducted as a proof of concept. The particle consists of the architecture identifier, three probe regions, and the remaining blank regions (Figure 4A, Figure S11, Table S1). Blank regions were used to avoid signal cross-talk caused by the manufacturing bias of the analyte regions; their signals could be disregarded in subsequent analyses. Different experimental objects were arranged in geometrically twisted regions to highlight the design flexibility and the analysis universality. The analyte probes include two domains: the target-specific domain for capturing the target analyte and a universal adapter domain for binding of the signal reporting probe that is identical for all analytes.^48,49^ The miRNA assay involved (i) target capture, (ii) binding of the universal adapter to the analyte probe-target complex under the action of T4 DNA ligase, and (iii) removal of unbound adapters due to their low affinity for direct binding to the analyte probe. Since the correspondence between signal reporting and specific target species was determined based on spatial information, single fluorophore-labeled universal adapters can be used to report multiple independent signals simultaneously. After the different reactions, red fluorescent signals highlighted the positive targets. Next, the corresponding reference particle was offered for signal analysis (Figure 4B, Figure S12). In each case, fluorescence was shown only in the region where the probe matched the target, indicating the specificity of the analysis (Figure 4C). These results validated the feasibility of multitarget detection on heterogeneous particles and suggest the potential of arbitrarily structured particles as integrated microsensor systems, accelerating the progress toward single-particle detection.

### AMPs as Multicellular Systems

**Figure 5:**
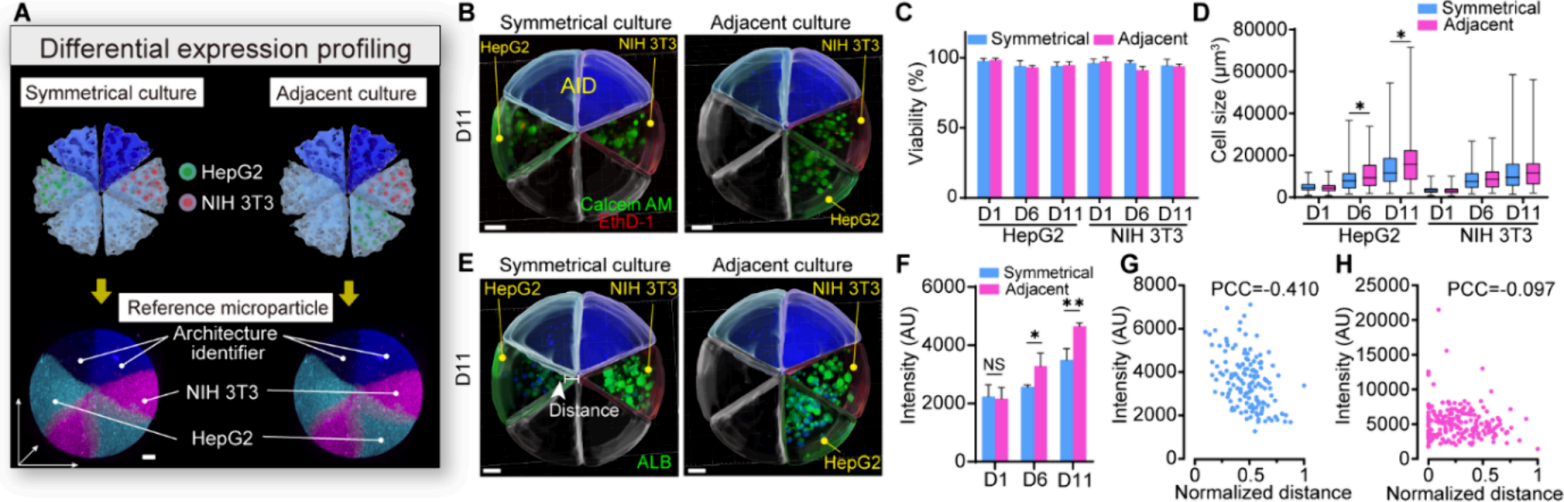
AMPs function as a structurally tunable multicellular system: multicell analysis on a particle. (A) Schematic of cell diversity under different culture modes. Comparison of symmetrical and adjacent cultures using HepG2 and NIH 3T3 cells. (B) Tracking cell growth after an 11-day culture (D11). The cells were stained with Calcein-AM/EthD-1. (C) Evolution of cell viability on particles after 1, 6, and 11 days (*n* = 3). (D) Size of the cell cluster after 1, 6, and 11 days (*n* = 88–172). (E) Immunostaining analysis of hepatocyte marker (albumin) and DAPI. Distance represents the shortest distance between each HepG2 cell and NIH 3T3 region. (F) Quantification of albumin signal of HepG2 during an 11-day culture period (*n* = 3). (G, H) Analysis of the relationship between albumin signal intensity of HepG2 cells and intercellular distance in symmetrical (G; *n* = 140) and adjacent (H; *n* = 196) cultures. Error bars indicate standard deviation. NS, not significant, *p < 0.05, **p < 0.01. (D) Mann–Whitney U-test. (F) Student’s *t* test. Pearson correlation coefficient, PCC. Scale bars, 100 μm.

Including the desired biological analytes within the adjustable architectures can further emphasize the versatility of the AMPs. As a proof of concept, AMPs were used to construct multicellular systems, where the cellular interactions (HepG2 and NIH 3T3) were fine-tuned by the different spatial assemblies (Figure 5A, Figure S13 and S14). Here, the cell-laden AMPs were designed with uniformly segmented compartments, enabling regular alterations of the cell layout. In this design, the architecture identifier consists of two adjacent compartments with differential fluorescence to ensure a unique alignment. Figure S15 shows that the preset particles were successfully obtained, and a high addressing accuracy (>0.92) of the cells was also achieved. This means that one can perform cell-specific analysis in a multicellular system without the use of cell labels. For example, cytochemistry could be used to assess the viability (Calcein AM/EthD-1 staining) of heterogeneous cells and hepatic functional expression (albumin staining). In terms of cell population scale, all cell types showed similar cell viabilities (above 91%) in both adjacent and symmetrical cultures over 11 days (Figure 5B, C, and Figure S16). However, a significant increase in the size of hepatocyte clusters was observed in adjacent cultures versus symmetrical cultures (Figure 5D). Additionally, compared to the symmetrical culture, albumin expression was significantly increased in the adjacent culture after a 6 day culture period (Figure 5E, F, and Figure S17). These results show close cultivation distances can result in better proliferation and functional performance of HepG2 cells. Furthermore, single-cell analysis details the heterogeneity of cellular response to the spatial arrangement. At the end of the 11-day culture, in the symmetric culture, the albumin intensity of HepG2 cells negatively correlated with the cell spacing (Figure 5G). In contrast, this trend was not observed in the adjacent culture, which indicates that microscale space arrangement influences the biological function of collective cell organization (Figure 5H).^50,51^ These results demonstrate that AMPs can be used to engineer microscale multicellular systems and perform label-free analysis.

## Conclusion

In summary, we report powerful and versatile approaches for the fabrication and analysis of AMPs, aiming to construct multiplexed assays on anisotropic particles at the single-particle level. The microfluidic approach enables the arrangement of desired experimental objects into a specified particle space, which is highly scalable and demonstrated by a series of complex designs. The proposed addressing strategy can be used for the accurate addressing of immobilized objects within the customized particles, allowing for the analysis of their in situ signals in a label-free manner without imposing restrictions on the patterns of subregions. The accurate addressing of different architectures, including regular and twisted compartmentalized layouts, confirms the reliability of the methodology and emphasizes its universality.

The AMPs can be designed as novel microscale carriers for targeting various multiplexed applications. The present article lays the foundation for this concept, highlights the outstanding contributions in biochemical sensing, and presents proof-of-concept biological studies. As a powerful demonstration, two embodiments (i.e., multiplexed bioassays and multicellular system construction) show the precise deployment, functional performance, and addressable analysis of molecules and cells within these particles. Also, these fundamental cases can inspire future applications of the hydrogel particles. For example, more single-microparticle-based detection tools can be developed by leveraging both existing and newly developed anisotropic particles. Designs may encompass integrating structure-driven mobility (e.g., magnetic response,^52^ self-orienting^53^) to improve reaction efficiency. These particles stand to benefit cost-effective clinical diagnostics. Looking ahead, we envision these particles as single-use detection units, akin to test strips. In cell culture applications, each particle could support a specific cell type or coculture system, enabling precise and localized studies. In addition, by replacing the encapsulated objects and materials, these particles can be further used to construct research-worthy microsystems such as artificial cells,^54^ organ simulations,^27,55^ and human model replications.^56^ Such innovations are also poised to deeply influence related fields, such as tissue engineering and drug screening.

Concentrating scientific “laboratories” on the microscopic scale brings opportunities to bridge life sciences and engineering in the context of moving toward flexibility, efficiency, and cost-effectiveness.^25,38,57^ The AMPs epitomized the “lab-on-a-particle” concept. We anticipate that the proposed methods will motivate microparticle systems to make breakthroughs in the microcosm.

## Supporting Information Available

Movie S1. The generation of artificial particles (MP4)
Movie S2. Object identification results of the pseudocolorized target particle (MP4)
Movie S3. Verification of identification accuracy by reactivating the fluorescence information on the target particle (MP4)
Detailed experimental methods and results (PDF)
