# Supplementary material for: Construction of Multiplexed Assays on Single Anisotropic Particles Using Microfluidics
Source: ACS Cent Sci. 2025 Jan 15;11(2):294–301. doi: 10.1021/acscentsci.4c02009 (PMC11868959; doi:10.1021/acscentsci.4c02009)
Supplement: Supplementary file 4 — oc4c02009_si_004.pdf [file oc4c02009_si_004.pdf]

Supporting Information

Construction of Multiplexed Assays on Single Anisotropic Particles Using Microfluidics

Zengnan Wu, † Yajing Zheng, † Ling Lin, ‡,* Gaowa Xing,† Tianze Xie,† Jiaxu Lin,† Xiaorui Wang,‡ and Jin-Ming Lin †,*

† Beijing Key Laboratory of Microanalytical Methods and Instrumentation, Key Laboratory of Bioorganic Phosphorus Chemistry & Chemical Biology (Ministry of Education), Department of Chemistry, Tsinghua University, Beijing, 100084, China.

‡ MOE Key Laboratory of Geriatric Nutrition and Health, Beijing Technology and Business University, Department of Bioengineering, Beijing Technology and Business University, Beijing, 100048, China.

Table of Contents

Methods S2
Supplementary Figures S8
Supplementary Movies S28
References S28

Methods

Materials

SU-8 2050 Negative photoresist and its developer were purchased from Microchem Corporation (Newton, MA, USA). PDMS prepolymers and initiators were procured from Dow Corning (Midland, MI, USA). 1H,1H,2H,2H-perfluorooctyl trichlorosilane and sodium alginate were obtained from Sigma-Aldrich (MO, USA). Calcium chloride was acquired from Yuanye (Shanghai, China). Fluorescent polystyrene beads of varying size and excitation/emission wavelengths (B200 (200 nm, 412/473 nm), G200 (200 nm, 468/508 nm), G0200 (2 μm, 468/508 nm), G0500B (5 μm, 468/508 nm), R0300B (3 μm, 542/612 nm), and R200 (200 nm, 542/612 nm)), Live/dead assay kit, Cell tracker™ Green CMFDA, Cell Tracker™ Deep Red were procured from Invitrogen (USA). Streptavidin-coated magnetic beads (200 nm) and DAPI staining solution were purchased from Beyotime (Shanghai, China). TBS buffer (150 mM NaCl, 10 mM Tris-HCl), Triton X-100, and normal saline were sourced from Solarbio (Beijing, China). X-Clarity mounting solution was purchased from Logos Biosystems (Korea). The cell culture essentials such as Dulbecco's Modified Eagle Medium (DMEM), fetal bovine serum (FBS), trypsin, penicillin, and streptomycin were supplied from Gibco Corporation (NY, USA). Water was purified by the Milli-Q ultrapure water system of Millipore. All oligonucleotides utilized in this work were synthesized and purified by Sangon Biotechnology (Shanghai, China). Any additional chemical reagents were of the highest grade available and were used as received.

Cell culture

HepG2 cells (cell line of human hepatocellular carcinoma) and NIH-3T3 fibroblasts (cell line of mouse embryonic fibroblast) were obtained from Cancer Institute & Hospital Chinese Academy of Medical Science. Both cell types were cultured in Dulbecco's Modified Eagle Medium supplemented with 10% FBS and 1% streptomycin and penicillin. The culture was maintained in a humidified incubator at 37 °C in 5% CO_2. The cells were seeded at 5×10^4 cells/cm2, sub-cultivated every 2 days, and the medium was refreshed every day.

Characterization

The production of the alginate particles was monitored in real-time using a digital microscope (Dino-Lite, Vidy Co., Ltd., China). The section images of particles were acquired with an inverted confocal laser scanning microscope (LSM880, Zeiss, Germany). The microparticle production frequency

was monitored using a VW-9000 high-speed camera (Keyence, Japan). To acquire the z-stack images, the samples were imaged by a light-sheet fluorescence microscopy (Zeiss, Germany). Each slice was illuminated from both the right and left sides of the sample, and a bilateral fused image was saved. The imaging time for each single microparticle is approximately 2 min.

Experimental set-up

The microfluidic chips were prepared using soft lithography.[1] The SU-8 2050 photoresist was spin-coated on a 75 mm silicon wafer using a spin-coater (KW-4A, Microelectronics Center, Chinese Academy of Sciences) at a speed of 1250 rpm for 90 s, resulting in a uniform thickness of 140 μm. After soft baking at 70 °C for 5 min, the silicon wafer underwent UV patterning through transparent photo masks. The obtained patterns were then again rebaked at 75 °C for 20 min and developed with the SU-8 developer. After developing the clear microstructures, the molds were treated with a silylation reagent (1H,1H,2H,2H-perfluorooctyl trichlorosilane) under a vacuum condition for 12 h. The degassed 10:1 mixture of PDMS and initiators was poured onto the molds and cured at 65 °C for 3 hours. Once the cured devices were prepared, PDMS stamps were peeled off from the mold pattern, and the channel inlets and the horizontal junction (the fluid convergence outlet) were made by using a precision punch with an X-Y platform. To fabricate the microfluidic channel composed of a single-layer chip, the PDMS replicas were tightly sealed to a glass substrate after oxygen-plasma (PDC-32G, Harrick Plasma, USA) activation of both surfaces and cured at 65 °C for at least an hour. The stacked chips were obtained by aligning multiple PDMS replicas under a microscope.

The droplet ejector was customized by two coaxial nested stainless-steel needles. The inner needle (26G) was fixed on the axis of the outer needle (17G), and its outlet shrinks 0.2 mm relative to the outlet of the outer needle. Two symmetrical air inlets are designed on both sides of the outer needle. To connect the microfluidic chip with the droplet ejector, the inlet of the inner needle was carefully inserted into the liquid outlet of the microfluidic chip, creating a seamless and leak-free junction for the entire fluid channel. The device assembly, including the chip and ejector, was securely fixed onto a glass substrate using hot melt adhesive to stabilize the components. This adhesive provided robust support, preventing any movement during operation. During droplet manufacturing, the microfluidic device was vertically secured in a fixture.

The solidification component includes a pair of sprayers containing a cross-linking solution ($CaCl_2$, 1 M) and a collecting bath ($CaCl_2$, 100 mM). The sprayer is oriented towards the droplet outlet to trigger rapid cross-linking.

Fabrication of hydrogel particles

Microparticles were continuously generated using the microfluidic device, which was connected with the 1 ml syringe (BD, USA) via polytetrafluoroethylene (PTFE) tube, connected with a gas-holder supplying high-purity nitrogen, and fixed on a vertical positioner. To produce a series of user-defined particles, various alginate solutions (1.5 w/v% in normal saline) were employed, each containing fluorescently labeled polystyrene beads (diameter in 200 nm) as visual objects. Green fluorescent beads were incorporated into the object fluid as model objects, while red fluorescent beads were added to the auxiliary fluid. We pumped alginate solutions as a dispersed phase into the fluid inlet and injected high-purity nitrogen through the droplet ejector. The liquid flow rates were controlled by syringe pumps (PHD 2000, Harvard Apparatus), and the nitrogen flow rate was adjusted by a rotameter. Concurrently, an aerosol of calcium chloride (1 M) was generated at the fluid outlet using a sprayer, initiating the cross-linking of the alginate. A collecting bath (100 mM $CaCl_2$) was used to collect and stabilize the newly formed particles. Using a similar approach, anisotropic particles were obtained by using corresponding microfluidic devices. The microstructure of the particles was visualized using an LSM880 inverted confocal laser scanning microscope. All 3D images of particles were acquired using the commercial Zeiss Lightsheet platform.

To fabricate both the target microparticles and their corresponding reference particles, the same microfluidic device was employed under consistent experimental conditions. The sole distinction was in the composition of the reference microparticles: heterogeneous fluorescent beads (200 nm in diameter) were utilized to replace the functional objects.

Alignment of target microparticle and reference microparticle

Alignment of the particles was conducted within a shared coordinate system. The image of the target particle was superimposed onto that of the reference particle, followed by a translation adjustment to align their geometrical center. Subsequently, the size of the reference particle was scaled to match the contour of the target particle, compensating for minor size discrepancies arising during fabrication. Next, the reference particle was fully segmented based on fluorescence boundaries and annotated in accordance with the predefined particle blueprint. We then proceeded to overlay the

architecture identifiers of the reference and target particles through rotation. Since both particles share an identical layout, this overlapping of architecture identifiers ensures the congruence of their spatial segmentation layouts. Consequently, the spatial attributes of the reference particles can be accurately projected onto corresponding elements within the target particles.

To enhance visual clarity and prevent fluorescence signal interference, we render the reference particles. Utilizing Imaris software, we engaged in interactive generation of a standardized, fully segmented reference particle, which entailed manual segmentation and 3D reconstruction (Figure S8). The resulting reference particle dataset comprised a 3D image stack with 500-800 optical slices, contingent upon its size. Parsing all slices under the supervision of experienced experts is labor-intensive, particularly for complex structural analysis. Therefore, to balance analytical precision and practicality, we routinely partitioned the 3D image stacks into 15-30 critical slices at uniform intervals. Segmentation was carried out slice by slice using the surface module in Imaris software, where distinct boundaries were marked between adjacent regions. Manually, we delineated each region on high-resolution images, guided by the fluorescence signals. Following this, we annotated the regions in accordance with the particle blueprint. Finally, 3D reconstruction was performed to amalgamate these segmented slices into a comprehensive segmentation volume, enabling the complete annotation of the reference particle.

Evaluation of addressing accuracy

To evaluate the accuracy of object addressing, we analyzed the fluorescent signals from object regions. These signals were categorized based on their distinct characteristics, including fluorescence color and particle diameter. The accuracy of addressing is defined as:

$$Accuracy = \frac{True\ signal\ (volume)}{True\ signal\ (volume) + False\ signal(volume)}$$

where true signals refer to those correctly identified, and false signals denote those incorrectly identified.

Preparation of miRNA probe-vehicle bioconjugates

The miRNA probe-vehicle bioconjugates were prepared by coupling the miRNA probes on streptavidin-coated magnetic beads (MBs, 200 nm in diameter). Initially, 100 μL of 10 mg/mL streptavidin-coated MBs suspension was washed three times with normal saline and re-dispersed in 400 μL of normal saline. An excess of biotin-modified miRNA probe was added to the above solution

and incubated for 2 h for the complete immobilization of miRNA probes on MBs. The resultant suspension was magnetically separated, washed three times with normal saline, and re-dispersed in 400 μL of normal saline for storage at 4 °C.

Fabrication of the particle hybridization system

The particle hybridization system was designed to load an architecture identifier and three types of miRNA capture probes into eight independent regions of a particle. The probe-loaded regions were isolated by blank regions. Target particles were produced by injecting varying 1.5% alginate solutions containing blue fluorescent beads or miRNA probe-vehicle bioconjugates into the microfluidic device at a flow rate of 1500 μl/h. The gas flow rate was maintained at 1.1 L/min. The concentrations of the spray solution and receiving solution were 1 M and 100 mM, respectively. Reference particles, with the same spatial layout but containing differently colored fluorescent beads, were fabricated similarly.

Performance of the particle hybridization system

For hybridization experiments, hydrogel particles were placed in separate centrifuge tubes. The particles were mixed with reaction solutions containing zero, one, two, or three targets. The hybridization systems were incubated in a TBS buffer at 55 °C for 2h. After the third rinse, the ligation solution (100 mM universal adapter, T4 DNA ligase 1000 U/ml, ATP (1 mM), and 1×NEB buffer) was added to the resultant particles. The mixture was incubated in the shaker at 37 °C for 1.5h. After post-incubation, particles were washed with saline for imaging.

Fabrication of the cellular interaction system

To design the cellular interaction system, we divided the particle into six independent regions to load a group of architecture identifiers, HepG2 cells, and NIH 3T3 cells. For microfluidic cell encapsulation, all devices were aseptically processed. We prepared 1.5% alginate solution containing 0.2% (w/v) blue fluorescent beads (diameter is 200 nm), 1.5% alginate solution containing 0.04% blue fluorescent beads (diameter is 200 nm), 1.5% alginate solution containing HepG2 cells with a concentration of 1×10^7 cell/ml, 1.5% alginate solution containing NIH 3T3 cells with a concentration of 1×10^7 cell/ml, and pure 1.5% alginate solution, respectively. Thereafter, synthesized functional precursors were simultaneously injected into a six-channel microfluidic device in a certain configuration at flow rates of 3500 μl/h. The gas flow rate was controlled by a rotameter at 1.0 L/min. The concentrations of the spray solution and receiving solution were 1 M and 100 mM, respectively.

Once cell-laden capsules were formed, they were collected and washed three times with cell culture media (DMEM) containing 5 mM $CaCl_2$. All particles were incubated in DMEM at 37 °C and 5% CO_2.

Cell labeling

Cells were harvested by centrifugation and aspiration of the supernatant. HepG2 and NIH 3T3 were incubated with 5 μM green cell tracker working solution (C7025, Invitrogen) and 1 μM red cell tracker working solution (C34565, Invitrogen), respectively, under growth conditions for 20 minutes. We then centrifuged the cells, removed the cell tracker working solution, and resuspended the cells gently in 1.5% alginate solution.

Assessment of cell viability

Cell viability was checked using the Live/Dead Cell Double Staining Kit. Cell-encapsulated particles were harvested by natural sedimentation, and followed by aspirating the supernatant. These cell-laden particles were incubated with Calcein AM/EthD-1 working staining solution under growth conditions for 25 minutes and later used for imaging. In cell-laden particles, the live cells show uniform green fluorescence (Ex/Em 488/515 nm), whereas dead cells produce red fluorescence (Ex/Em 570/602 nm). The viability of each cell type is defined as:

$$Viability(\%) = \frac{Volume\ of\ live\ cells}{Volume\ of\ live\ cells + Volume\ of\ dead\ cells}$$

Immunofluorescence staining

The cells cultivated within the hydrogel particles were fixed in a 4%(w/v) paraformaldehyde for 25 min and permeabilized with 0.1% triton X-100 for 15 min. After treatment with 5%(v/v) bovine serum albumin for 1 h, the samples were incubated with rabbit polyclonal anti-albumin (1:100, PA5-89332, Invitrogen) at 4 °C overnight, followed by incubation with secondary antibodies that were labeled with Alexa Fluor 488 (A32731, Invitrogen) at room temperature for 1 h. The samples were counterstained with 4',6-diamidino-2-phenylindole for 5 min. To acquire high-resolution information, the samples were immersed in the transparent reagent (X-Clarity hydrogel solution) for 5h at room temperature, followed by 3D imaging. Transparent reagents can make tissues transparent, thus achieving clear and bright 3D fluorescence imaging at any tissue depth.[2] The z-stack images were imaged using the Lightsheet microscope.

Supplementary Figures

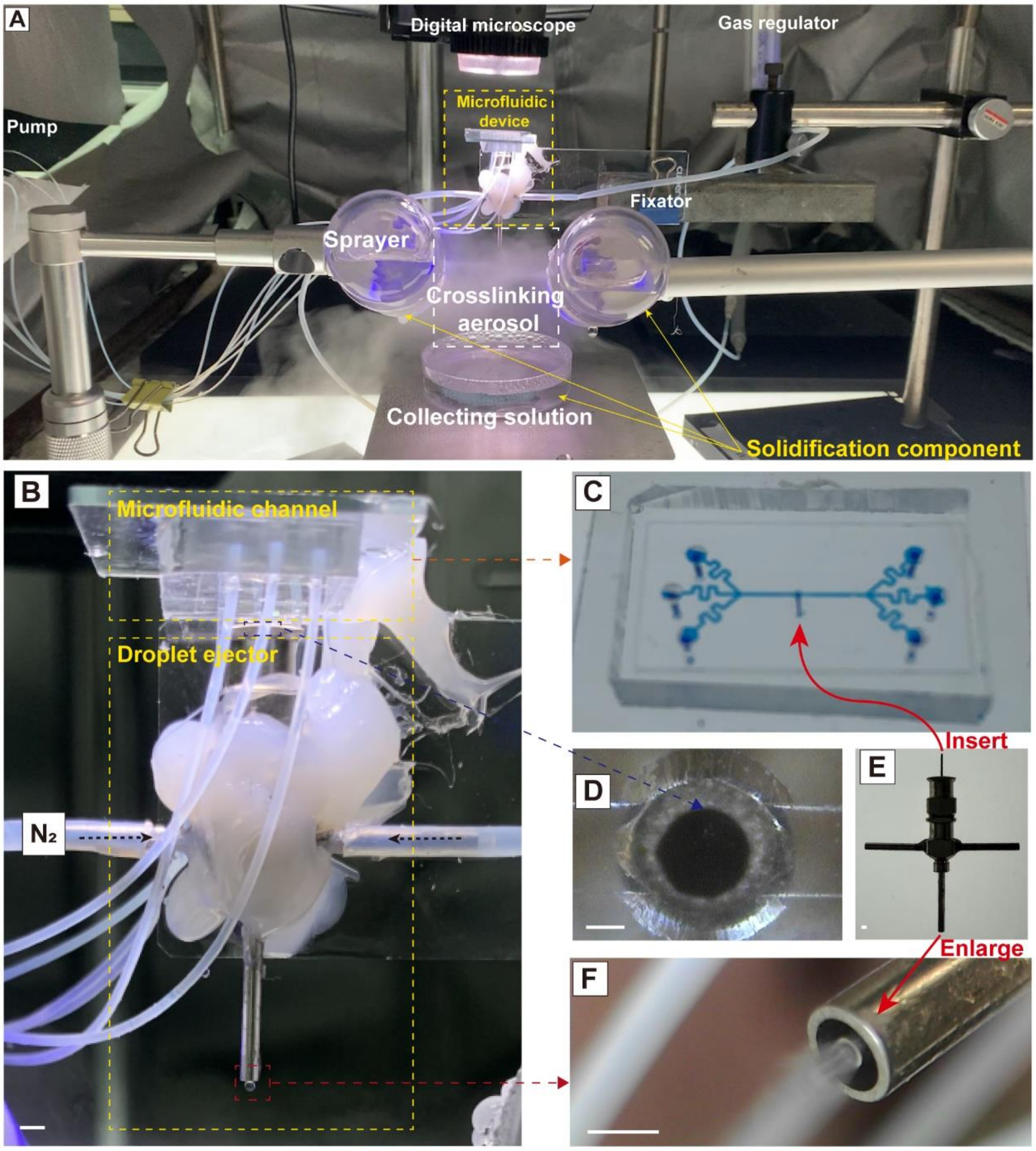

Figure S1. Schematic view of the experimental equipment. (A) An image of the experimental device for customizing particles. An image of the experimental device for customizing particles. The microfluidic device consists of a microfluidic channel, a droplet ejector, and a solidification component. The equipment was fixed in the vertical direction. A pair of sprays containing cross-linking solution were symmetrically arranged below the droplet outlet. The receiving solution containing a cross-linking solution was located directly below the device for collecting and stabilizing alginate particles. (B) An image showing that the microfluidic channel and the droplet ejector are connected by a junction. Scale bar, 2 mm. (C) An image of the chip-based microfluidic channel with a channel width of 250 μm. (D) The digital microscope image of the junction. Scale bar, 100 μm. (E) An image of the droplet ejector. Scale bar, 1 mm. (F) Snapshot of the droplet formation. Scale bar, 1 mm.

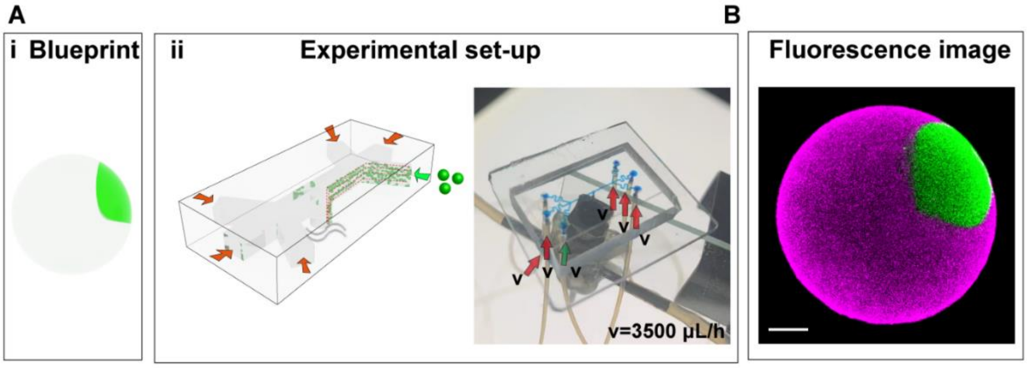

Figure S2. Fabrication of the artificial particles. Model objects (fluorescent nanoparticles, 200 nm) are arranged in the edge region of the particles. (A) (i) Particle blueprint. (ii) Schematic of the experimental setup. (B) Cross-section images of the obtained particles. Scale bars, 100 µm.

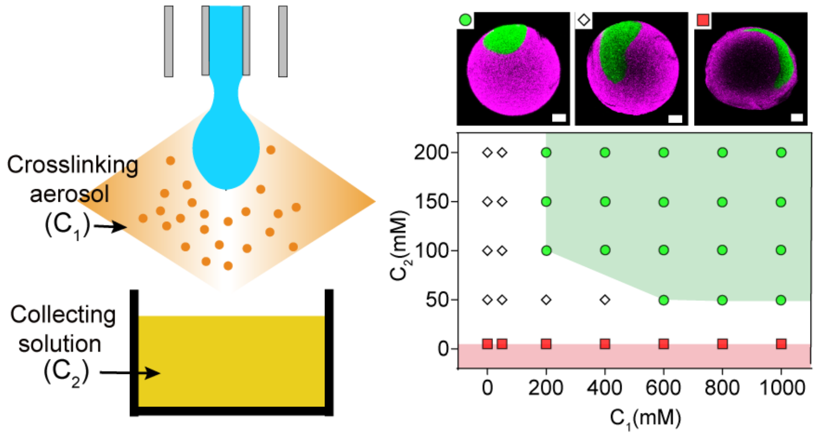

Figure S3. Optimal calcium chloride concentration for the stable formation of pre-design particles. C1 is the calcium concentration of the cross-linking aerosol; C2 is the calcium concentration of the collecting solution. Green circles represent ideal particles. White diamonds represent particles with disturbed regional boundaries. Red squares represent particles with dispersed contours. Scale bars, 100 μm.

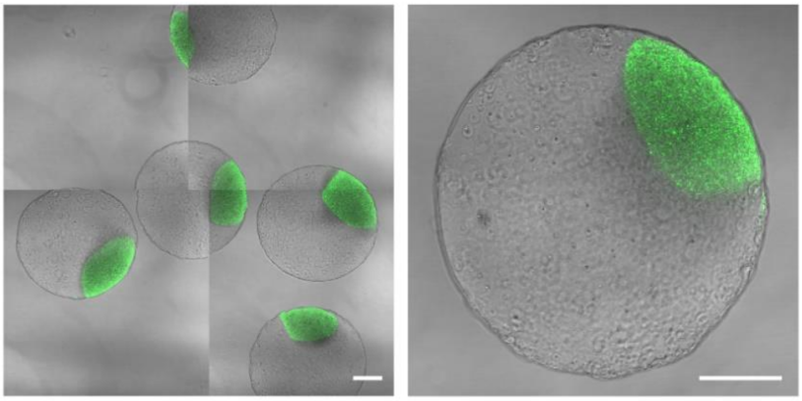

Figure S4. Optical image of the obtained particles. Scale bars, 200 μm.

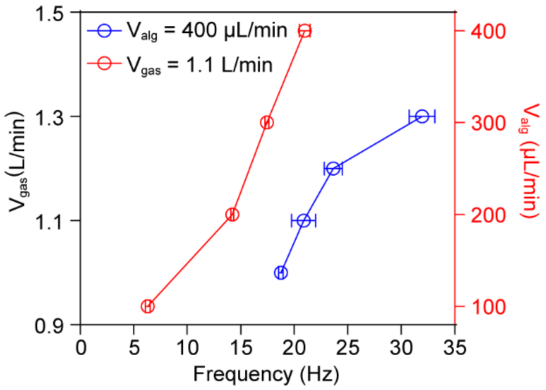

Figure S5. Droplet production frequency. Effect of gas flow rate and alginate flow rate on droplet generation frequency (n = 3). Error bars represent the standard deviation.

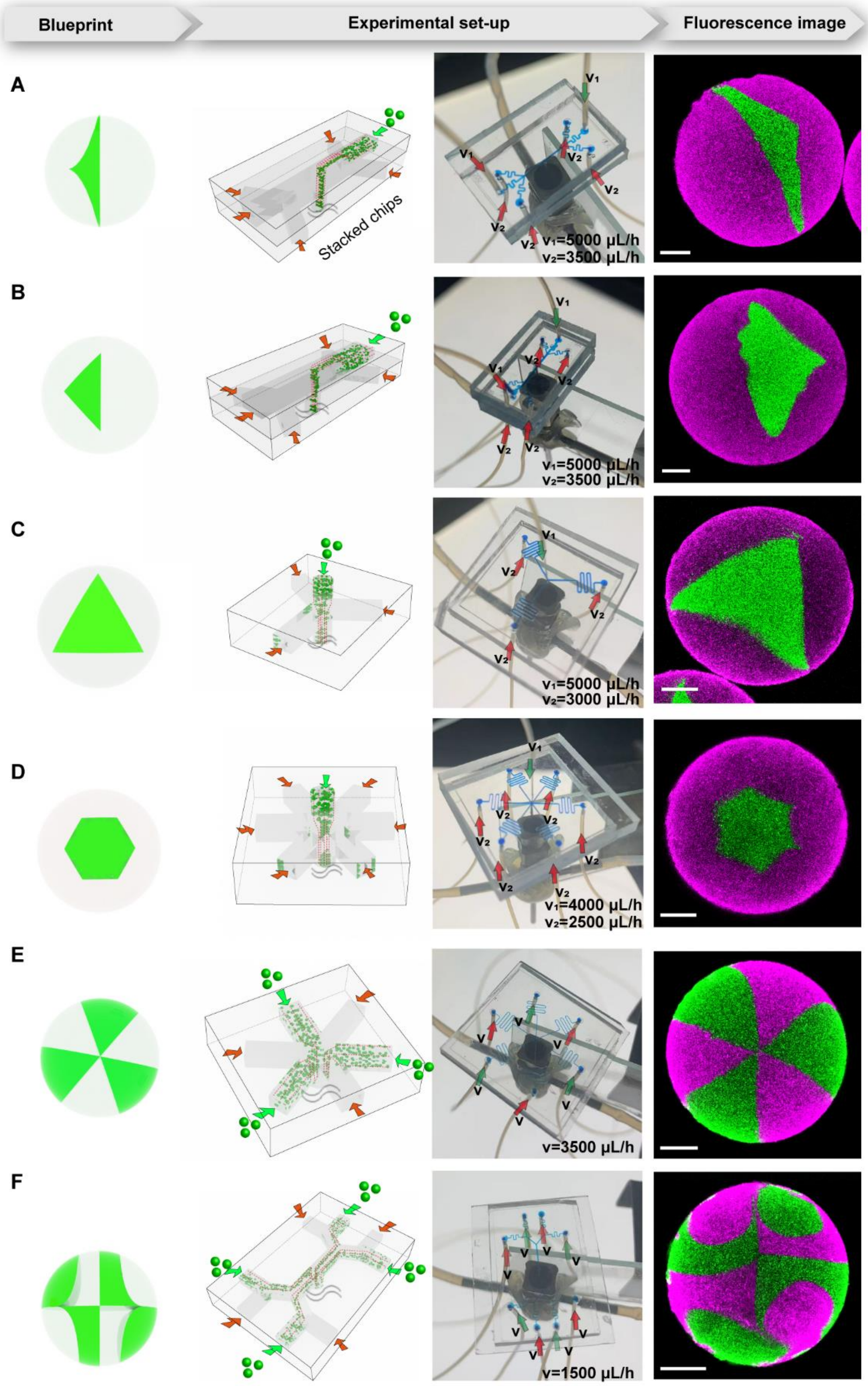

Figure S6. Model objects are arranged in the user-defined space within the particles. (A-F) Various object layouts are formed: the expended hemispheric triangle region (A), the contracted hemispheric triangle region (B), the central triangular region (C), the central hexagonal region (D), the "clover" region (E), and the irregular region (F). Model objects are green fluorescent beads (200 nm). Scale bars, 100 μm.

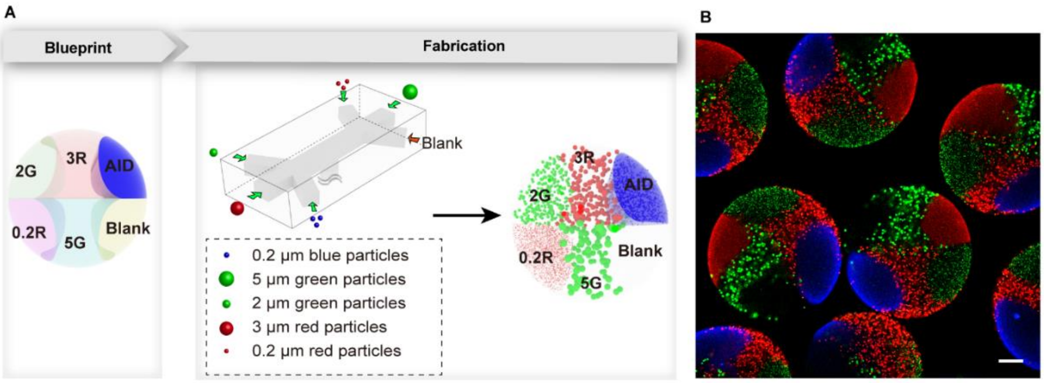

Figure S7. Fabrication of the model particles. (A) Schematic of model particle architecture. Regional abbreviations: AID, architecture identifier; 2G, 2 µm green beads; 5G, 5 µm green beads; 0.2R, 0.2 µm red beads and 3R, 3 µm green beads. The AID region is filled with 0.2 µm blue fluorescent beads. (B) Confocal images of the obtained model particles. Scale bars, 100 µm.

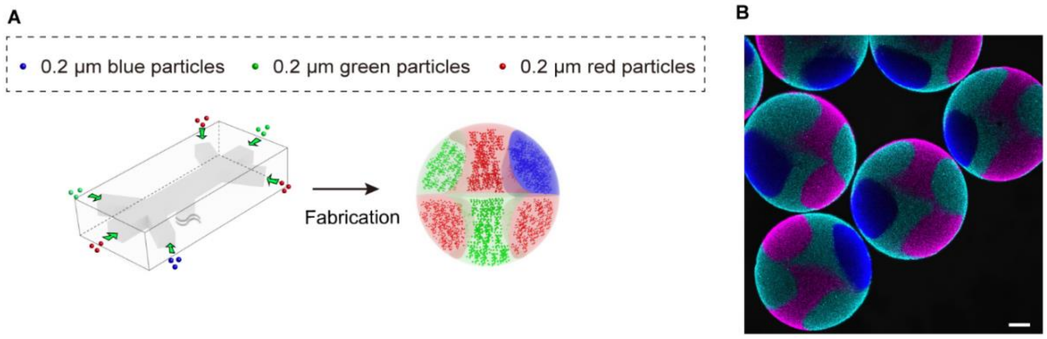

Figure S8. Fabrication of the reference particle. (A) The reference particles were obtained from the corresponding microfluidic device. The fluorescent identifier (fluorescent particles of 0.2 μm diameter) was used to visualize and segment the region boundary. (B) Confocal images of the reference particles. Scale bars, 100 μm.

Generation of the reference microparticle

After obtaining 3D image of the reference microparticle, its images are subjected to a series of operations, including segmentation, annotation and reconstruction (Figure S9A). The data set of the obtained reference particle is a 3D image stack (Figure S9B). On each typical slice, sharp boundaries were displayed between adjacent regions. Each region could be outlined according to the guidance of the fluorescence signal. Regional annotation was then carried out according to the microparticle blueprint (Figure S9C). Finally, we performed reconstruction to combine the segmentation slices into a segmentation volume, thereby obtaining a standardized, fully segmented reference microparticle (Figure S9D).

To assess the reliability of the spatial segmentation, five participants were invited to perform this procedure (Figure S9E-G). The results showed that the volume variation of the segmented regions ranges from 2.1% to 4.4%, suggesting the stable generation of the reference microparticle.

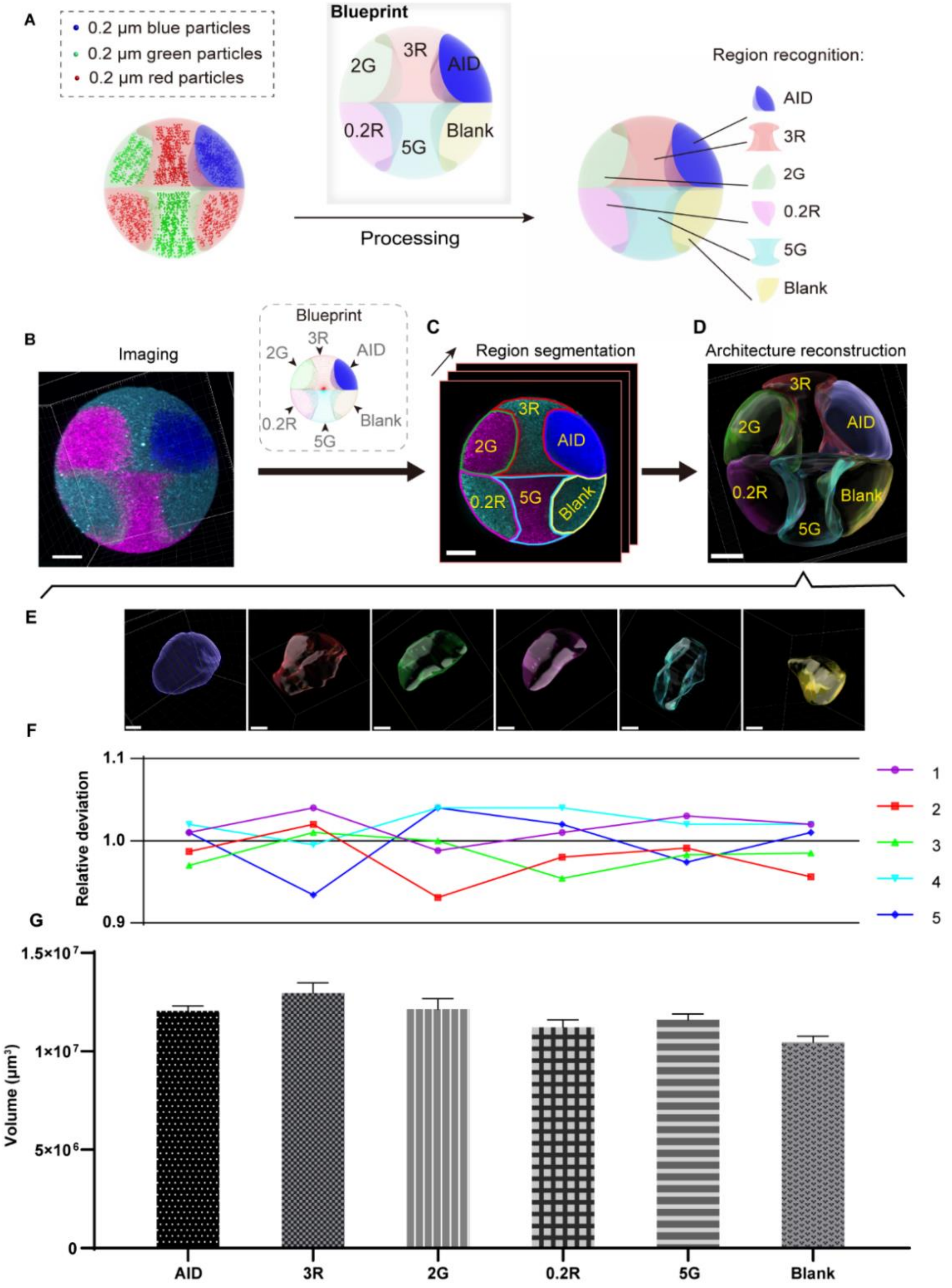

Figure S9. Workflow of standardizing the reference particle. (A) Schematic of the processing of the reference particle. (B) Fluorescence images of the reference particle. (C) Region segmentation and annotation of the microparticle based on fluorescent identifiers. Visual identifiers show regional boundaries to support segmentation. The particle is spatially divided into 2G, 5G, 0.2R, 3R, architecture identifier, and blank regions, which correspond to the particle blueprint. (D) Architecture reconstruction of the reference particle. Regional abbreviations: 2G, 2 μm green beads; 5G, 5 μm green beads; 0.2R, 0.2 μm red beads and 3R, 3 μm green beads. Reproducibility of segmentation of the reference particle: (E) Typical images of the regions that constitute the reference particle. (F) Segmentation bias between 5 operators. The geometric mean volume of each region is set to 1, and all volumes are scaled accordingly. The crossing lines in this image indicate that there is no systematic bias

between operators. (G) The volumes of the typical regions derived from five participant statistics (n = 5). Error bars represent the standard deviation. Scale bars, 100 μm.

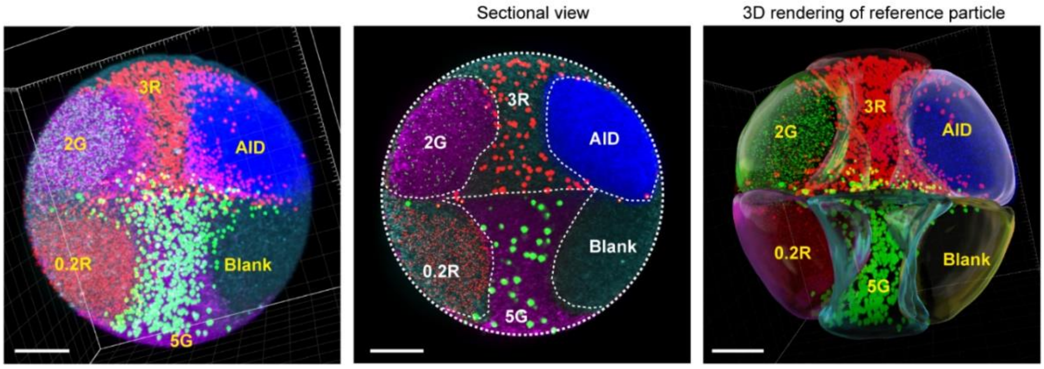

Figure S10. Verification of object identification by reactivation of original fluorescence. Scale bars, 100 μm

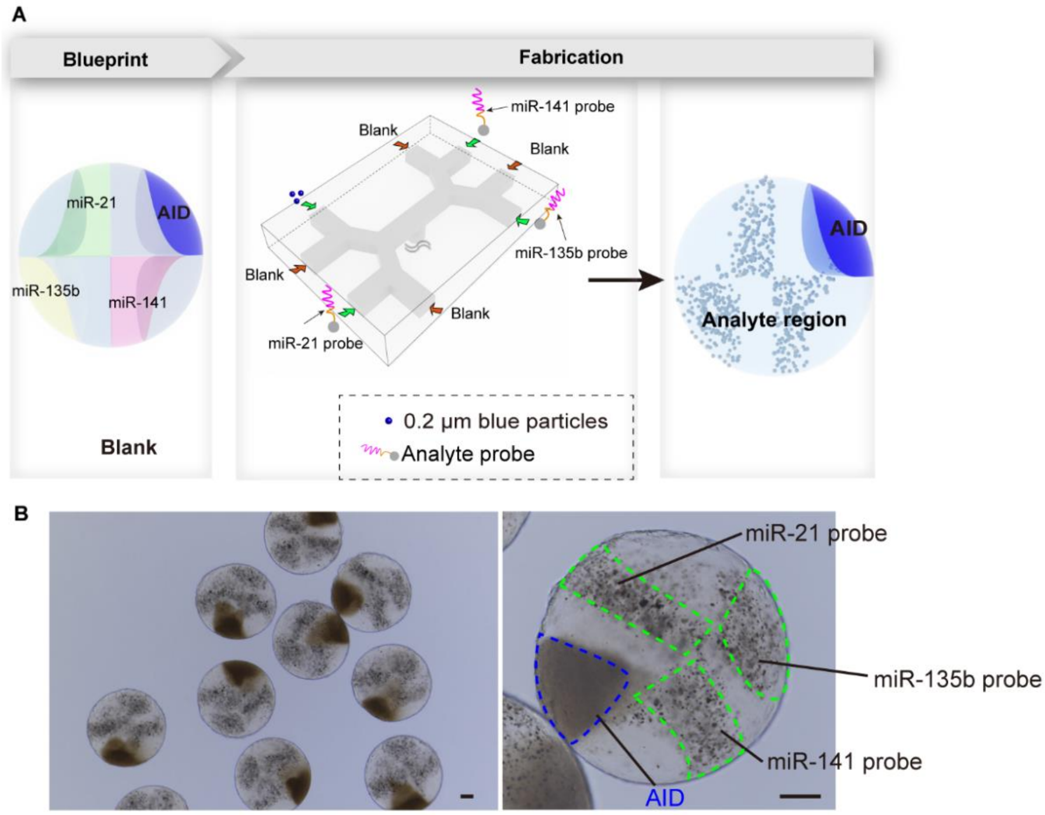

Figure S11. Microparticles for multiplexed detection. (A) Schematic of particle manufacturing. (B) Bright-field images of the obtained particles. Scale bars, 100 μm.

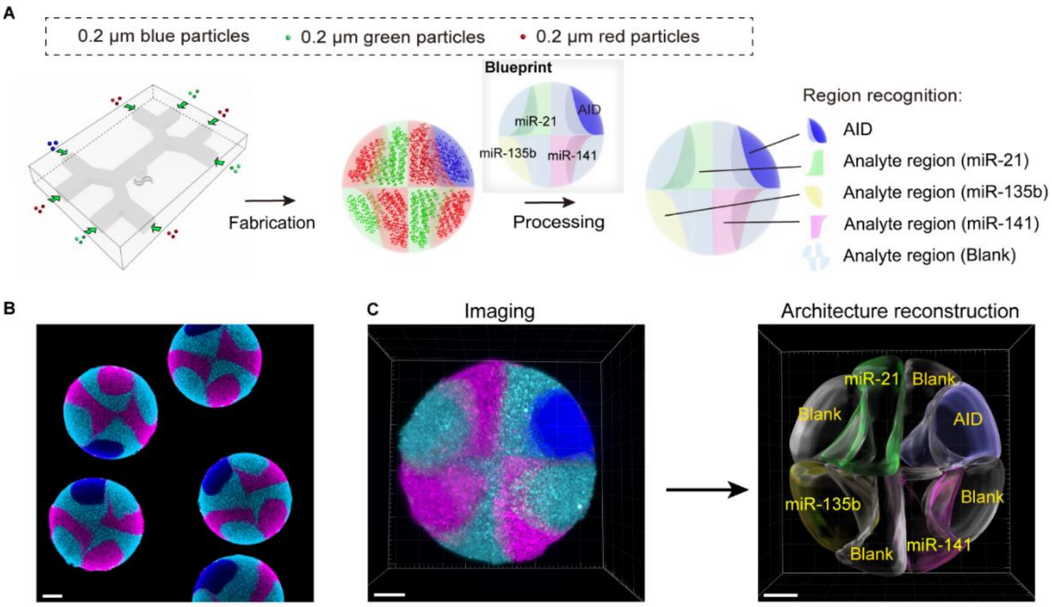

Figure S12. Generation of reference particles for multiplexed detection. (A) Schematic of the generation of the reference particles. (B) Fluorescence images of the obtained reference particles. (C) Images of the reference particles. Scale bars, 100 μm.

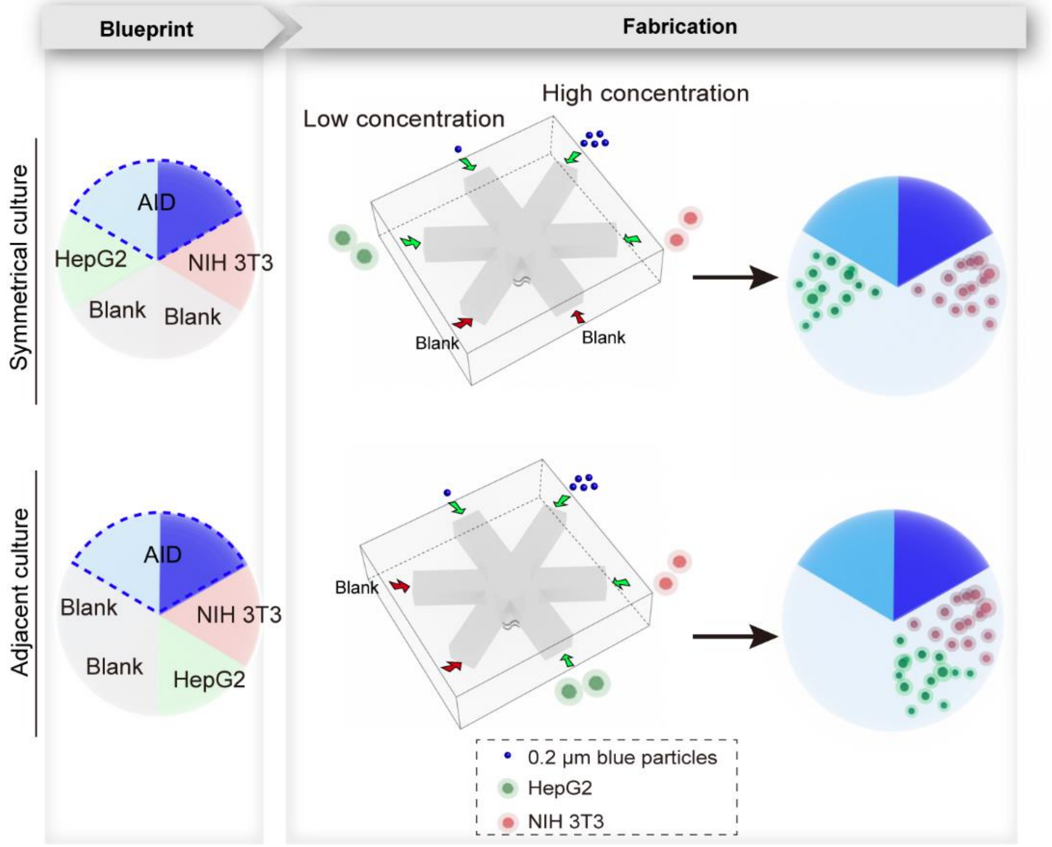

Figure S13. Microparticles as a cell analysis tool. Cells (HepG2 and NIH 3T3) were carried out adjacent and symmetrical co-cultures, respectively.

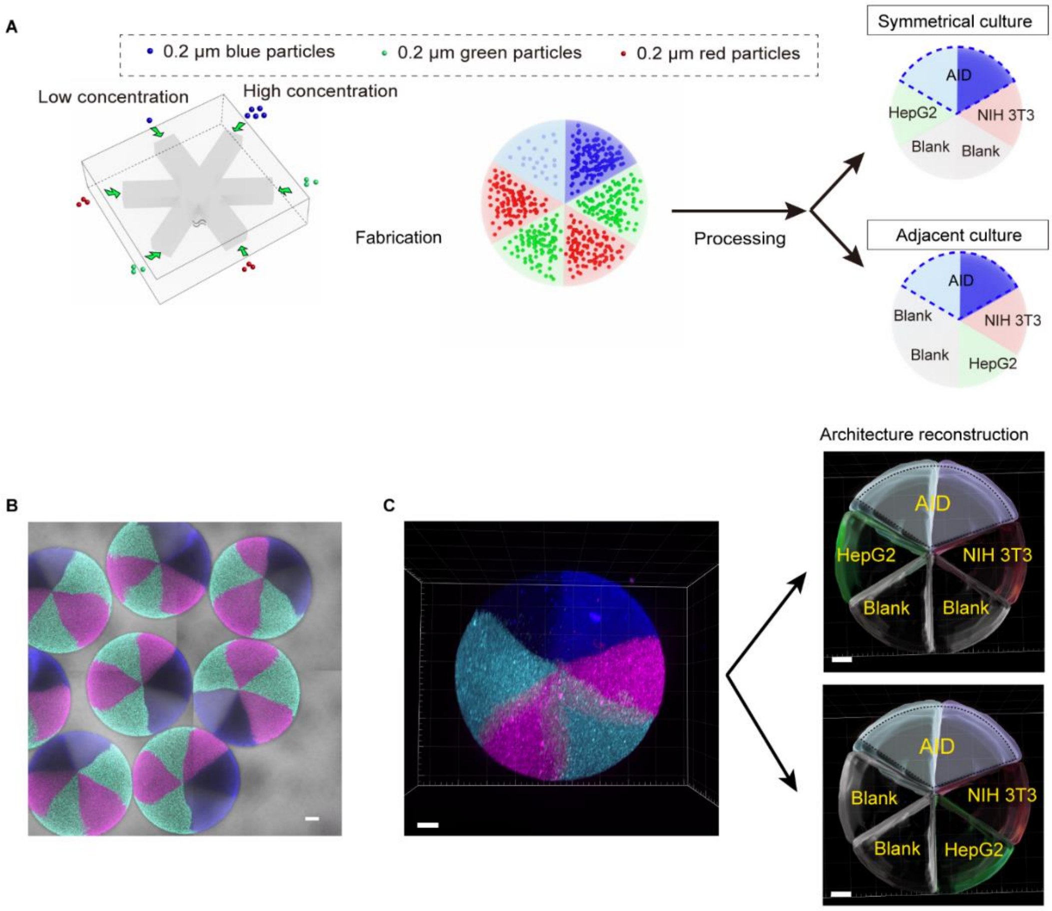

Figure S14. Generation of reference particles for cell analysis. (A) Schematic of the generation of the reference particles. (B) Fluorescence images of the obtained reference particles. (C) Images of the reference particles. Scale bars, 100 μm.

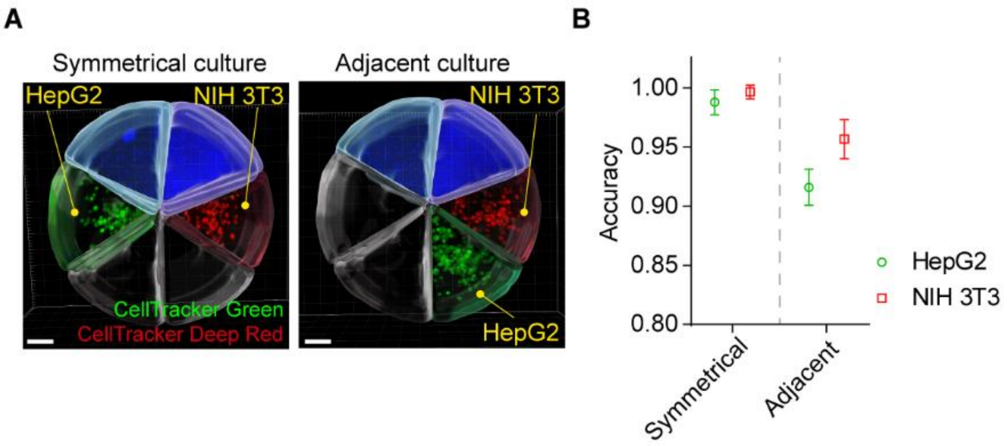

Figure S15. Validating the accuracy of cell addressing. (A) Tracking specific cell types by labeling HepG2 and NIH 3T3 cells with green and red cell trackers, respectively. (B) Quantification of the accuracy of cell addressing (n = 3). Error bars represent the standard deviation. Scale bars, 100 μm

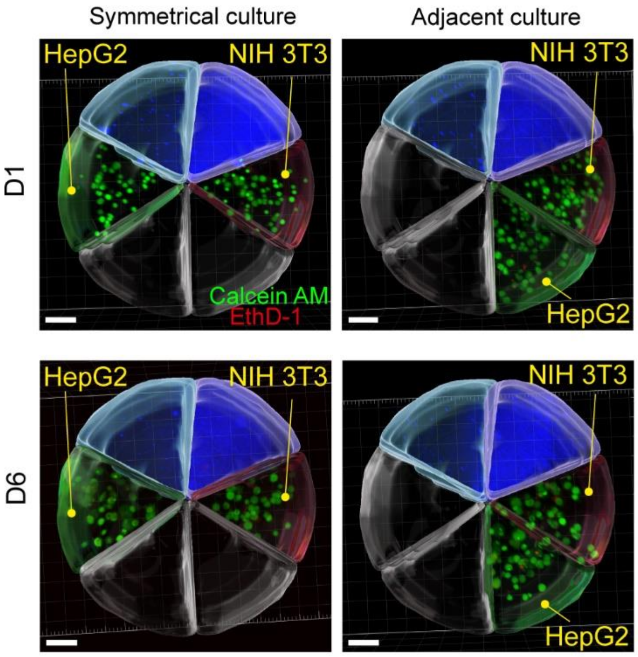

Figure S16. Cell viability test. Representative images of particles stained for LIVE/DEAD after 1 and 6 days. Cell viability is characterized by the Calcein AM/Ethd-1 staining kit. Scale bars, 100 μm.

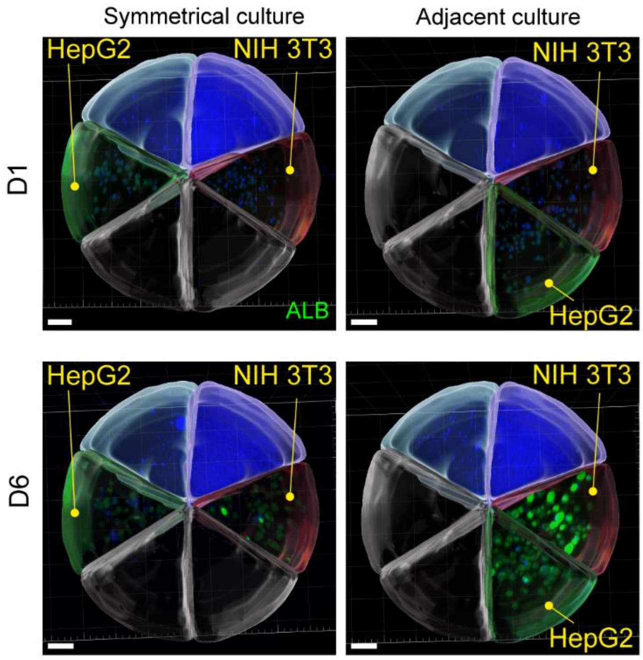

Figure S17. Cell function evaluation. Immunofluorescence staining is used to reveal the albumin expression in different culture configurations. Representative images of particles stained for albumin (ALB) and DAPI. Scale bars, 100 μm.

Table S1. The sequence used in this work.

Name		Sequence (5`-3`)
miR-21	Analyte probe	UAGCUUAUCAGACUGAUGUUGA
	Reporting probe	Biotin-GATATATTTTATCAACATCAGTCTGATAAGCTA-Inverted dT
miR-135b	Analyte probe	UAACACUGUCUGGUAAAGAUGG
	Reporting probe	Biotin-GATATATTTTACCATCTTTACCAGACAGTGTTA-Inverted dT
miR-141	Analyte probe	UAUGGCUUUUCAUUCCUAUGUGA
	Reporting probe	Biotin-GATATATTTTATCACATAGGAATGAAAAGCCATA-Inverted dT
Universal reporting probe		Phos-TAAAATATAT-Cy3

Supplementary Movies

Movie S1. The generation of artificial particles.

Movie S2. Object identification results of the pseudo-colorized target particle.

Movie S3. Verification of identification accuracy by reactivating the fluorescence information of the target particle.

References

(1) Xia, Y. N.; Whitesides, G. M. Soft lithography. *Angew. Chem. Int. Ed.* **1998**, *37* (5), 550-575.

(2) Chung, K.; Wallace, J.; Kim, S.-Y.; Kalyanasundaram, S.; Andalman, A. S.; Davidson, T. J.; Mirzabekov, J. J.; Zalocusky, K. A.; Mattis, J.; Denisin, A. K.; et al. Structural and molecular interrogation of intact biological systems. *Nature* **2013**, *497* (7449), 332-337.
